# Structure-guided forcefield optimization

**DOI:** 10.1002/prot.23013

**Published:** 2011-02-15

**Authors:** Yifan Song, Michael Tyka, Andrew Leaver-Fay, James Thompson, David Baker

**Affiliations:** 1 Department of Biochemistry, University of WashingtonSeattle, Washington 98195; 2 Howard Hughes Medical Institute, University of WashingtonBox 357370, Seattle, Washington 98195

**Keywords:** forcefield optimization, hydrogen bond potential, rotamer library

## Abstract

Accurate modeling of biomolecular systems requires accurate forcefields. Widely used molecular mechanics (MM) forcefields obtain parameters from experimental data and quantum chemistry calculations on small molecules but do not have a clear way to take advantage of the information in high-resolution macromolecular structures. In contrast, knowledge-based methods largely ignore the physical chemistry of interatomic interactions, and instead derive parameters almost exclusively from macromolecular structures. This can involve considerable double counting of the same physical interactions. Here, we describe a method for forcefield improvement that combines the strengths of the two approaches. We use this method to improve the Rosetta all-atom forcefield, in which the total energy is expressed as the sum of terms representing different physical interactions as in MM forcefields and the parameters are tuned to reproduce the properties of macromolecular structures. To resolve inaccuracies resulting from possible double counting of interactions, we compare distribution functions from low-energy modeled structures to those from crystal structures. The structural and physical bases of the deviations between the modeled and reference structures are identified and used to guide forcefield improvements. We describe improvements resolving double counting between backbone hydrogen bond interactions and Lennard-Jones interactions in helices; between sidechain-backbone hydrogen bonds and the backbone torsion potential; and between the sidechain torsion potential and Lennard-Jones interactions. Discrepancies between computed and observed distributions are also used to guide the incorporation of an explicit Cα-hydrogen bond in β sheets. The method can be used generally to integrate different sources of information for forcefield improvement.

## INTRODUCTION

High-resolution protein structures provide an invaluable source of information for forcefield testing and improvement. The parameters of knowledge-based energy functions are entirely derived from protein structures. With the assumption that distributions of interatomic distances and other protein structure properties are independent and Boltzmann distributed, the underlying energy is obtained simply by computing the negative logarithm of the observed distributions.^1^ However, the distributions are far from independent, and this approach also has the disadvantage of neglecting the large body of knowledge on the physical chemistry of interatomic interactions. 2 Molecular mechanics (MM) forcefields on the other hand derive most parameters from experimental and quantum chemistry data on small molecule data rather than proteins. 3–8 The total system energy is expressed as the sum of terms with clear physical origins. The applicability and transferability of such forcefields to macromolecules has been demonstrated by showing that native structures are at least metastable in molecular dynamics (MD) simulations and by reproducing vibration spectroscopic data. 6, 8 However, these tests only probe the accuracy of a forcefield in the neighborhood of the native state; longer time scale simulations are now starting to test forcefield accuracy over a larger range of conformational space. 9, 10 The Rosetta forcefield, like MM forcefields, expresses the total system energy as the sum of physically interpretable terms but uses protein structure data both to refine parameters and to model contributions that are difficult to obtain by other means. 11 For example, the Rosetta forcefield supplements Lennard-Jones and implicit solvation terms used in MM forcefields with protein structure-derived sidechain and backbone torsion potentials.

Double counting of the same physical interaction by two different forcefield terms can result in overall forcefield inaccuracies. It is not straightforward to systematically correct this problem. In MM forcefields, the backbone torsion potential, combined with the rest of the forcefield, may incorrectly bias the balance between helix and sheet structures. This problem has been addressed by comparing molecular dynamics simulations to NMR coupling and relaxation data and quantum chemistry calculations^12^, ^13^ and adjusting the torsional potentials accordingly. For residue-resdiue knowledge-based potentials, an iterative improvement approach has been described 14 that compares computed and experimentally observed minima.

In this study, we use an iterative approach to detect and remedy problems resulting from double counting in forcefields based on the properties of energy minima distributed throughout conformational space. Systematic structural comparisons between the X-ray crystal structures and refined Rosetta models generated both near and far from their native state are used to track down errors in the standard Rosetta forcefield. The physical origin of the errors are identified and used to guide correction of the individual forcefield terms and to explicitly remove double counting.

## METHODS

### Crystal structure dataset

A set of high-resolution crystal structures was used as the reference for atom-atom distance, angle, and torsion distribution calculations. X-ray crystal structures of 1257 proteins were selected using PISCES^15^, ^16^ with resolution better than 1.5 Å, crystallographic *R* factor better than 0.3, and maximum sequence identity of 25%. Water and ligands were removed.

### Energy landscape generation

Energy landscape calculations and characterization are described in detail by Tyka *et al*.^17^ In brief, low-resolution models spanning a broad range of RMSD to the native structure (0∼20 Å) are generated using the Rosetta *ab initio* folding protocol 18 for 110 proteins. These 110 proteins are selected to include a variety of secondary structural elements and structural features. They include 24 all-alpha, 29 all-beta, and 57 alpha-beta proteins. Among these proteins, 17 of the structures bind a ligand, 60 are oligomeric, and 37 contain disulfide bonds.

The Rosetta full atom refinement protocol^18^, ^19^ is then applied to search for local minima in the vicinity of each low-resolution model with either the standard or the optimized energy function. For each protein, 100,000 all-atom refined models are generated, and the models are then placed into bins based on their RMSD to the native structure (bin width 0.5 Å). In each RMSD bin, the 20% of structures with lowest energies are collected for distribution calculations. This ensures that the distributions reflect contributions spanning a large range of structures not just conformations near the native structure.

The energy gap between native and non-native structures is taken to be the (average energy of the lowest 1% of structures that are less than 2 Å RMSD from the native structure) minus the (average energy of the lowest 1% of structures that are greater than 4 Å from the native structure).

### Distribution calculation

Atom-atom radial, angular, and dihedral distribution functions were collected for the 1257 protein crystal structure dataset and the low-energy computed structures for the 110 selected proteins described in the previous section. The low-energy Rosetta models from different RMSD bins were pooled together in the distribution calculation. Backbone atom-atom radial distributions were determined between all backbone atom pairs for each secondary structure type (secondary structure was designated as α-helix, β-strand, or loop as determined by the DSSP algorithm^20^). From crystal structures, there are a total of 2.7 × 10^5^ helix, 0.6 × 10^5^ β-sheet, and 1.0 × 10^5^ loop residues. Atom pairs less than 10 Å apart were collected for 1.2 × 10^6^ helix–helix residue pairs, 0.7 × 10^6^ strand–strand pairs, and 0.9 × 10^6^ loop–loop pairs. In each iteration of Rosetta modeling, distributions were computed from 1.3 × 10^7^ helix, 1.0 × 10^7^ β-sheet, and 1.3 × 10^7^ loop residues. The atom–atom radial distribution function is the average density of an atom2 at a distance *r* from atom1,


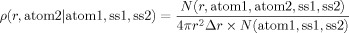
(1)

where the bin width Δ*r* = 0.1 Å bin,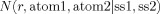 is the smoothed [Eq.(2)] counts of atom pairs (atom1 and atom2) at distance *r*with secondary structure ss1 and ss2, and 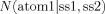 is the sum of *N*(*r*,atom1, atom2|ss1, ss2) over all atom2 types.

The counts 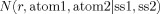 were smoothed using a Gaussian kernel:


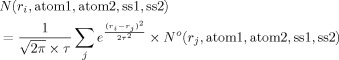
(2)

where 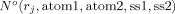 is the total counts at *r*_j_ before smoothing, and the bin width and variance τ are 0.1 Å.

Distributions were also determined for angles between hydrogen-bonding atoms. Two angles are measured: Θ, the angle formed by the donor heavy atom–donor proton–acceptor atom triplet, and Ω the angle formed by the donor proton–acceptor atom–acceptor base atom triplet.^21^

A total of 5.7 × 10^4^ helix and 3.7 × 10^4^ β-sheet backbone hydrogen bonds were collected from crystal structures, and 5 × 10^6^ were collected from low-energy Rosetta models in each iteration of forcefield optimization. Angular distributions were calculated using:


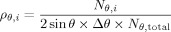
(3)

where Δθ, the bin width, is 5°, *N*_θ, total_ is the total number of hydrogen bonds, *N*_θ, *i*_ is total counts in the angle bin θ_*i*_. Counts were first smoothed using Gaussian kernel smoothing with variance 5°.

Backbone dihedral distributions (Ramachandran distributions) were collected for each residue type in each secondary structure. In the reference crystal structure set, for a given residue type and secondary structure, the numbers of residues collected for the distribution calculations range between 2,000 and 5,000. In the low-energy Rosetta models, the numbers of residues collected for each residue type and secondary structure combination range between 2 × 10^5^ and 4 × 10^5^. Counts were binned into two-dimensional 10° bins in φ and ψ and then smoothed using a kernel width of 10°.

For sidechain distributions, we followed previous work^22^–^24^ and classified sidechain conformations into discrete rotamer bins based on their χ angles and tabulated statistics separately by 10° backbone φ/ψ bins. Only rotamers with less than 20% accessible surface area are counted in the distribution. The rotamer definitions and the treatment of the terminal χ angle for Asn, Asp, Gln, Glu, His, Phe, and Tyr (5 or 10 degree bins) were as in the 2008 Dunbrack rotamer library. 24 Gaussian kernel smoothing was applied to both the backbone dependency and the terminal χ angles for the sidechains listed above; for the remaining fully rotameric sidechains, no smoothing was applied.

### Identification of inaccurately modeled features

We assess the forcefield at each iteration by computing the difference in the logarithms of the distributions of each of the above features between the reference crystal structures and low-energy Rosetta structures.


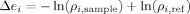
(4)

where ρ_*i*,sample_ and ρ_*i*,ref_ are the smoothed Rosetta model and crystal structure distributions. Large differences between the crystal structure distribution and modeled structures indicate potential areas for forcefield improvement. As we wish to focus improvements in frequently sampled regions and to reduce noise in the log-difference due to small counts, we scale the difference by the average frequency with which each bin is populated:


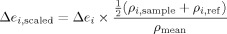
(5)

where ρ_mean_ is the Boltzmann average probability over all bins in the crystal structures:


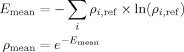
(6)

We focused subsequent analysis on regions where the scaled log difference between the distributions was greater than 1.

### Forcefield optimization

The regions where large differences are found between Rosetta modeled structures and crystal structures were analyzed, and the physical origin of the discrepancies were identified to determine the potential term that needs improvement. Identification involved inspection of the structural contexts of the discrepancies and analysis of the potential terms contributing to the Rosetta structure distributions.

Once the potential term requiring correction was identified, an iterative approach was applied to update the form of the potential guided by the difference between Rosetta models and crystal structures. For the Cα hydrogen bond potential (described below), Ramachandran potential and rotamer potential, the collected distribution function has the same dimension as the potential function, and therefore, the log difference between the Rosetta model and crystal structure distributions [Eq.(5)] can be directly subtracted from the potential term at each iteration:


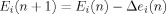
(7)

where *E*_*i*_ (*n*) is the potential function used in the nth iteration, and Δ*e*_*i*_ (*n*) is the log difference in Eq.(5) at the *n*th iteration. The new potential is then used for a new round of full atom refinement to generate a new set of near-native and non-native structures. Iterations of structure model generation, distribution calculation, and potential correction continue until Δ*e*_*i*_ (*n*) is below 0.5 for all bins. It took four iterations for the Cα hydrogen bond potential to reach convergence, 12 iterations for Ramachandran potential, and eight iterations for rotamer potential. A similar iterative approach was used previously to improve a simple pairwise residue-residue potential.^14^

The hydrogen bond potential is the sum of energy terms based on hydrogen bond distance and angles,


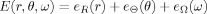
(8)

where *r* is the distance between the donor proton and acceptor atom, θ is the hydrogen bond angle formed by donor heavy atom–donor proton–acceptor atom, and ω is formed by donor proton–acceptor atom–acceptor base atom as described earlier.^21^ At each iteration, the potential forms of *e*_Θ_ and *e*_Ω_ are maintained, but the peak of the potential is shifted:


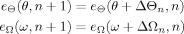
(9)

where ΔΘ_*n*_ is the difference in the peak position between the input *e*_Θ_ (θ) potential and the sampled distribution in the *n*th iteration. A similar correction is applied to *e*_Ω_ (ω). The update of a single parameter rather than the entire potential function reduced over-fitting artifacts.

The new Cα hydrogen bond potential was defined simply as a distance-dependent interaction between Hα and O. The starting guess at the potential was simply the log of the crystal structure Hα—O distance distribution function, and then the iterative approach described above was applied to optimize the potential until the modeled Hα—O radial distribution function matched that of crystal structures.

### Independent benchmark test

An additional benchmark test was applied to a dataset independent from the 110 proteins used for the optimization. This benchmark test uses 55 protein structures from the CASP8 experiment.^25^ The HHSearch protocol 26 was used to generate alignments to the pre-CASP8 database of template structures. Complete models based on those alignments were generated using Rosetta loop modeling. 27, 28 Then, the Rosetta full atom refinement protocol 18, 19 was applied to search for low-energy models with either standard or the optimized energy function. For each protein, 10,000 all-atom refined models were generated.

## RESULTS

**Figure 1:**
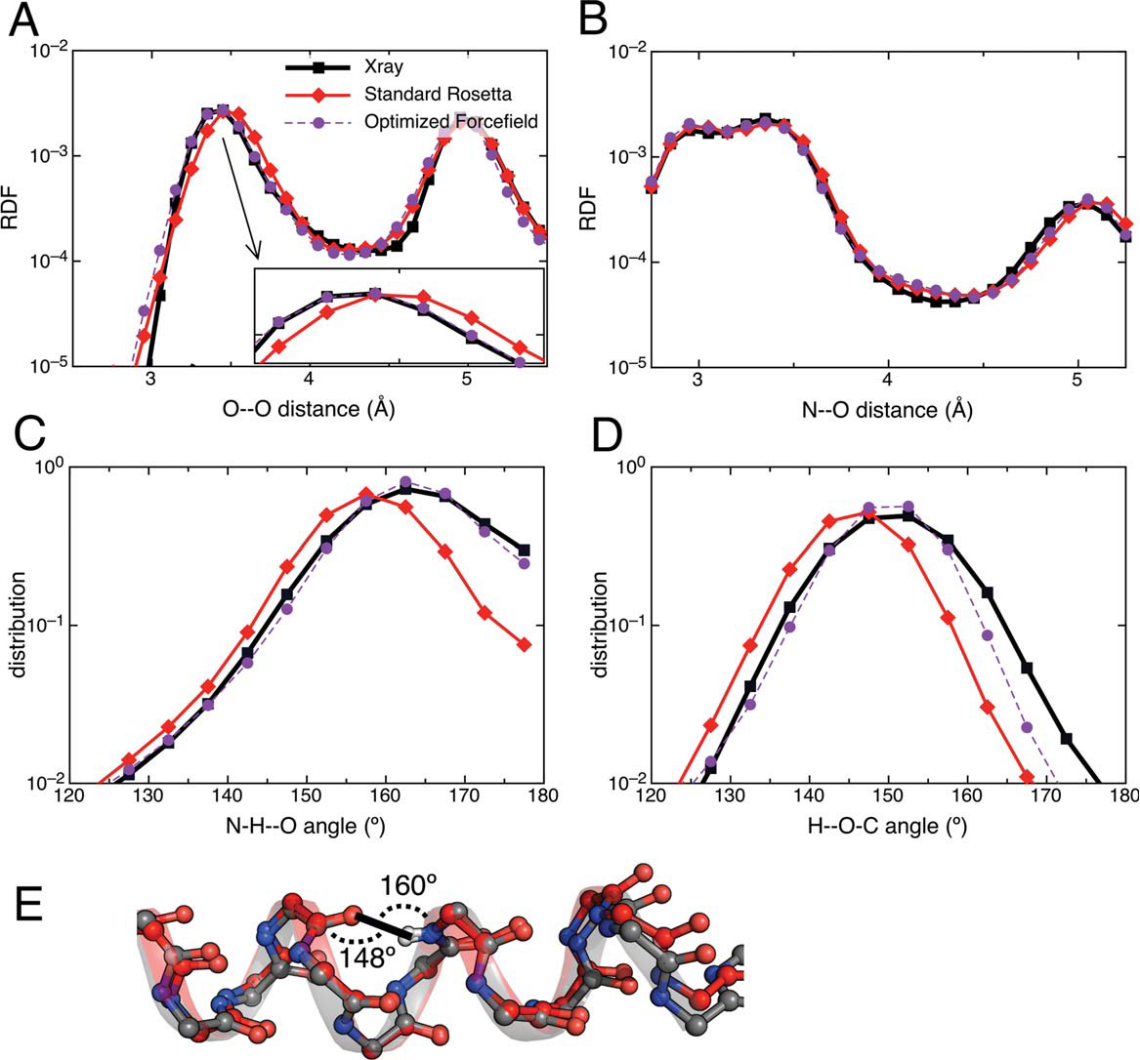
Comparison of helix backbone geometry in crystal structures and low-energy computed structures. Radial distribution function between (**A**) atom O and O (**B**) N and O; angular distribution of (**C**) hydrogen bonded N—H**···**O and (**D**) H**···**O—C. Black: distributions from crystal structures, red: Rosetta models with the standard forcefield, and magenta: Rosetta models with the optimized forcefield. (**E**) Illustrations of helix structures found in X-ray structures (gray) and Rosetta models with standard forcefield (magenta). The measured hydrogen bond angles in the crystal structure are highlighted.

**Figure 2:**
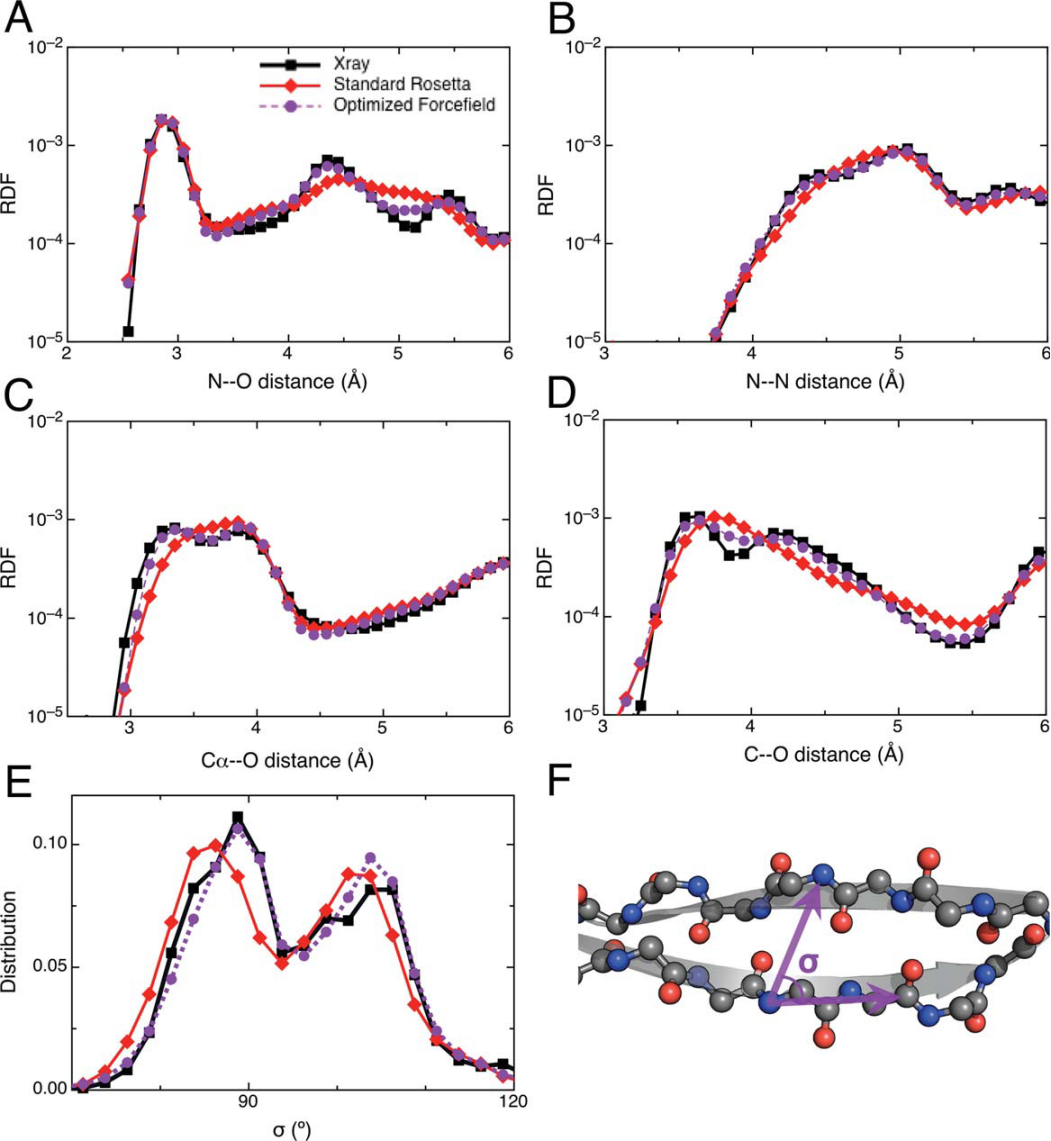
Comparison of β-sheet geometry. Radial distribution function of β-sheet backbone atoms are calculated between (**A**) N and O, (**B**) N and N, (**C**) Cα and O, (**D**) C and O, and (**E**) distribution of the angle between strand dimers in adjacent sheets (σ).^36^ Black: distributions from crystal structures, red: Rosetta models with standard forcefield, and magenta: Rosetta models with the additional nonpolar hydrogen bond potential. (**F**) Illustration of σ, which is measured as the angle between the N to C vector from adjacent residues on the same β-strand and the N to N vector from the pairing strands.

As described in the Methods section, we generated large ensembles of conformations sampling local minima throughout the energy landscape for a set of 110 proteins of known structure. From these ensembles, we computed distributions of interatomic distances and bond torsion angles. These distributions were compared with those observed in high-resolution protein structures, and the atom pairs and torsions for which the distributions in the computed energy minima differed significantly with crystal structures were flagged. For the majority of atom pairs and torsion angles, the distributions match quite closely; several examples are shown in Figures1 and 2.

Significant discrepancies between the distributions in crystal structures and computed energy minima likely reflect errors in the forcefield. Inspection of these discrepancies suggested that the majority of the errors reflect double counting of the same physical chemistry by two different energy function components. Other discrepancies were traced to missing energetic contributions. These errors and their resolution are described in the following sections.

### Helix backbone hydrogen bonding

The distribution of the hydrogen bond distance between the backbone nitrogen and the carbonyl oxygen is similar in crystal structures and low-energy Rosetta models (Fig.1). However, the distribution of distances between pairs of carbonyl oxygen atoms differs between the two sets of structures. As shown in Figure 1(A), the distribution peak at 3.4 Å in crystal structures is shifted to 3.6 Å in Rosetta models. This peak is due to pairs of backbone oxygens in residues adjacent in the sequence [Fig. 1(E)]. These atoms primarily interact with each other in the Rosetta forcefield via Lennard-Jones interactions. The helix hydrogen bond angular distributions for the angles formed by the atom triplets (N—H)—O and H—(O=C) also differ between Rosetta energy minima and crystal structures. The peak for the (N—H)—O angle is shifted from 162° in crystal structures to 157° in Rosetta models, and the H—(O=C) angle is shifted from 152° to 145°.

To identify which energy terms contribute to the observed shifts of the peaks in the hydrogen bond angle distributions, low-energy models were generated with all Rosetta energy terms up- or down-weighted individually. When the Lennard-Jones repulsion term was changed, changes in hydrogen bond angle peaks were the most significant, suggesting the shift of the peak is likely due to the coupling between the Lennard-Jones term and hydrogen bond energy: as neighboring oxygens are pushed apart by steric repulsion, the angles formed by hydrogen bonded atoms becomes smaller [Fig.1(C,D)]. This effect originates from the protein structure-derived hydrogen bond potential for helices in Rosetta. Sidechain hydrogen bond geometries in protein structures agree closely with those expected from MP2 QM energy landscape calculations, but helix hydrogen bonds differ considerably because of the constraints presented by protein backbone geometry.^29^ The error is that the protein structure-derived potential already incorporates the effects of steric and other interactions, so that including both the helical structure-derived hydrogen bond potential and the Lennard-Jones potential results in double counting.

We correct for this double counting by subtracting the contribution of the Lennard-Jones interactions from the angular dependence of the helix backbone hydrogen bond using the iterative procedure described in the Methods. The corrected potential is now more favorable for N—H—O angles near 180° and H—O—C angle near 165°. An additional ensemble of structures was generated with the corrected potential, and the N—H—O angle peak was found to be 162° and the H—O—C angle peak at 150°, matching crystal structures. In addition, the peak O—O distance shifted to 3.4 Å, agreeing with crystal structures as well. This improved agreement in a feature not directly controlled by the modification of the potential suggests that the correction strategy is on the right track. There are remaining errors in the region H—O—C > 160° [Fig.1(D)] with the corrected potential, because corrections are applied only to the position of the peak; the remaining difference in the distribution is in regions with very low populations.

### β-sheet Cα—O hydrogen bond

The first peak in the backbone β-sheet N—O or N—N distance distribution is very similar in crystal structures and low-energy rosetta models. In addition, the backbone β-sheet hydrogen bond angle distributions in Rosetta models match quite closely to those in crystal structures. Thus, the geometry immediately around the β-sheet hydrogen bond appears to be well modeled in Rosetta. However, there is a discrepancy in the atom pair distributions in β-sheet structures that is evident in the second peak of the N—O and Cα—O distributions. The Cα—O distribution shows a peak at 3.3 Å in the crystal structures (Fig.2), which is somewhat flattened and pushed further away in Rosetta models. The N—O distribution in crystal structures has a clear peak between 4 and 5 Å [Fig. 2(A)], whereas Rosetta models have a more flattened distribution.

We considered the possible origins of this discrepancy. There have been many suggestions that nonpolar hydrogen bond interactions contribute to protein structural features.^30^–^32^ In antiparallel β-strands, the shearing of the neighboring strands leads to nonlinear CO—NH hydrogen bonds. 33, 34 This was proposed to be due to the nonpolar hydrogen bond interactions. 35 The propensity of right hand twist in β-sheets has also been suggested to arise from Cα hydrogen bond interactions. 30, 35 The peak in the Cα—O distribution in crystal structures could reflect contributions of such hydrogen bonds, or it could be a secondary consequence of β-sheet packing and other well-understood contributions to protein energetics. However, the Rosetta forcefield explicitly models β-strand hydrogen bonding and accounts for the major contributions to protein energetics, hence the former explanation seemed more likely.

We tested the incorporation into the forcefield of a simple distance-dependent nonpolar hydrogen bond potential between the backbone carbonyl oxygen and Hα. The starting guess at the form of the potential was based on the logarithm of the distance distribution between the pair of atoms in crystal structures, normalized such that the potential is −0.5 at 3.0 Å and goes to zero at 3.6 Å. This starting guess was then refined by application of the iterative correction protocol described in the methods. The converged O—Hα potential has a depth about 40% the strength of the polar hydrogen bond.

With the incorporation of the nonpolar hydrogen bond term, the β-sheet geometry of low-energy Rosetta models closely matches crystal structures. The radial distribution of O to Cα now has the clear first peak found in crystal structures, and more significantly (since it is not explicitly enforced by the new term), the peak in the N—O distribution between 4 and 5 Å is now also clearly distinguishable. The registration of paired β-sheets also becomes more similar to crystal structures. As shown in Figure2(E), the distribution of σ, the angle between strand dimers in adjacent sheets^36^ used in the Rosetta low-resolution energy function, is shifted relative to crystal structures with the standard Rosetta energy function. With the incorporation of the nonpolar hydrogen bond, β-sheet registration in Rosetta models closely matches that in crystal structures. This agreement is again significant, because the σ distribution improvement is not forced directly by the nonpolar hydrogen bond term; instead the improvement in registration and the N—O distribution suggests β-sheet geometry as a whole has improved, and further that the nonpolar hydrogen bond contributes to the observed geometry in protein structures.

This example illustrates a strength of our approach—we make inferences not from the distributions observed in crystal structures, as for example traditional knowledge-based potentials—but from the differences between these distributions and those of models generated with a forcefield representing the major contributions to protein energetics. Differences as in this case are a strong argument for missing physical chemistry.

### Backbone torsion potential

**Figure 3:**
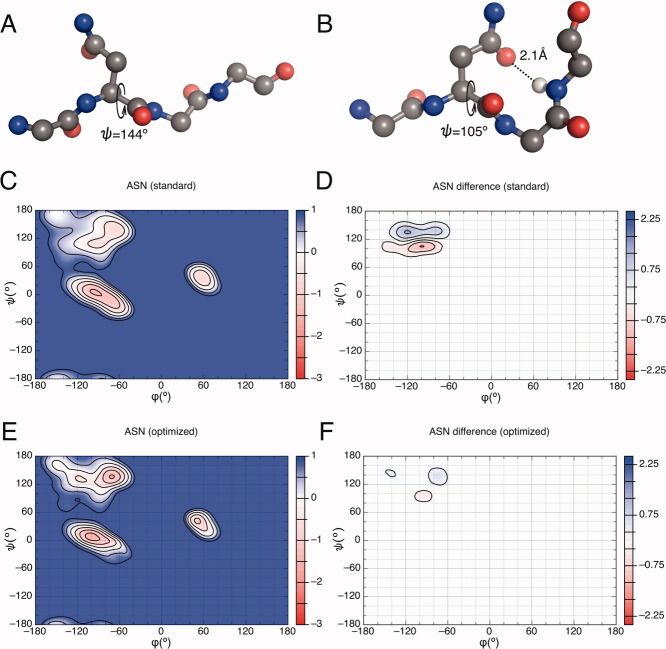
Backbone torsion angle distribution for asparagine. Frequently observed conformations of Asn are illustrated in (**A**) when backbone ψ is > 130° and (**B**) when backbone ψ is < 120°. (C) Ramachandran potential of Asn in the standard Rosetta forcefield, and (E) after optimization. The differences in backbone torsion distribution between Rosetta models and the reference distribution are shown for (**D**) the standard Rosetta forcefield and (**F**) with the optimized Ramachandran potential. The color scale shows the scaled differences in Ramachandran distributions as calculated by Eq.(5).

**Figure 4:**
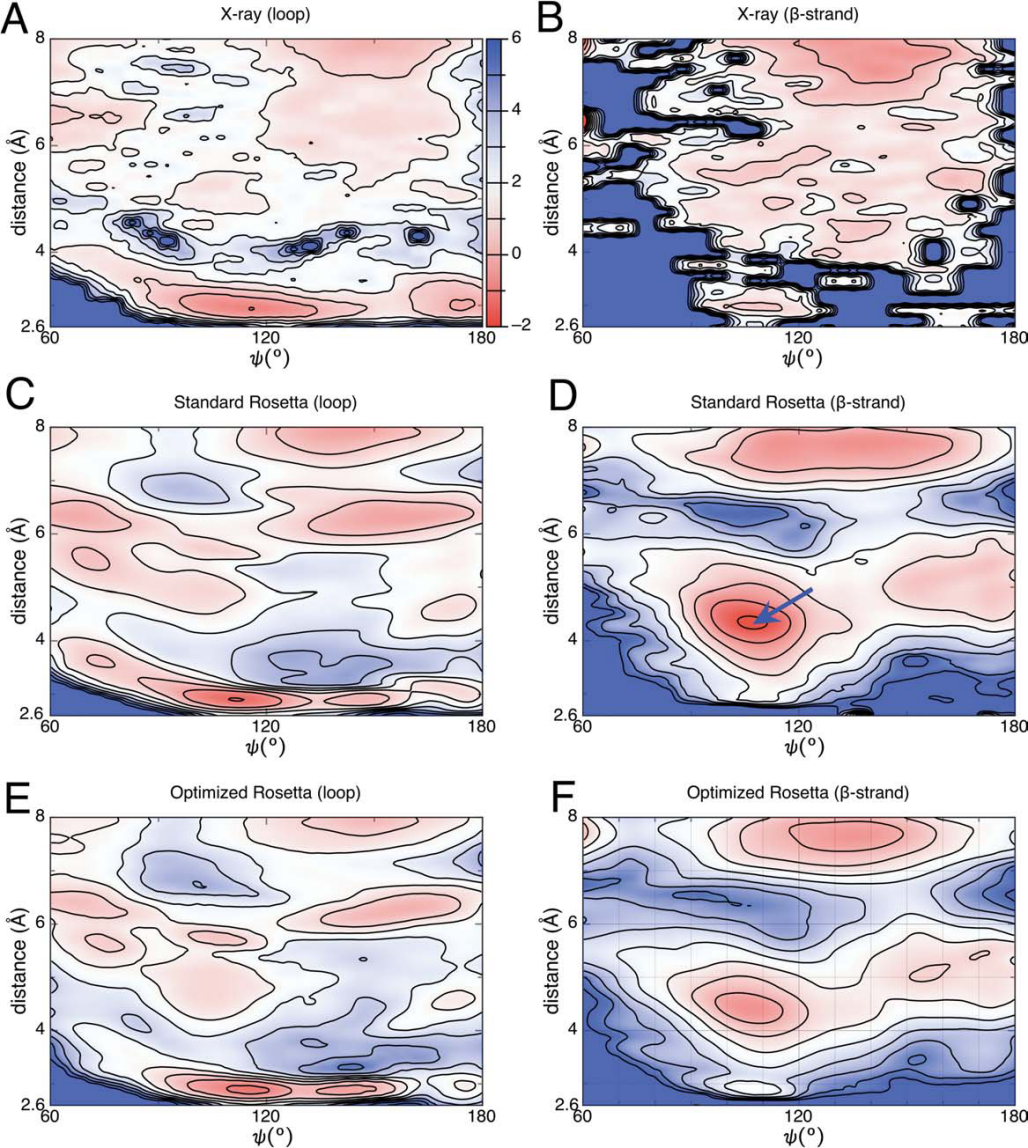
Coupling between backbone torision angles and sidechain-backbone hydrogen bond. Distributions of Asn backbone ψ (*x* axis) and atom-pair distance between Asn Oδ1 and N of residue *i* + 2 (*y* axis) in (**A**,**B**) crystal structures, (**C**,**D**) Rosetta models with the standard forcefield, and (**E**,**F**) Rosetta models with optimized forcefield for (A,C,E) loop and (B,D,F) β-strand secondary structure. The low energy regions in the loops (A,C,E) show that Rosetta stabilizes the conformation in Fig. 3B as in crystal structures, where the hydrogen bond restrains the backbone ψ to lower than 120°. The blue arrow in (D) highlights the artefact with the standard Rosetta forcefield; the hydrogen bond is not formed, yet the backbone ψ is still favored to be lower than 120°. The sampling of this region is much reduced with the optimized forcefield in (F). The color scale shows the minus log of probability of a given ψ and Oδ1—N distance bin, offset by *E*_mean_ [Eq.(6)].

For each amino acid in each secondary structure (helix, strand, turn), we collected statistics on the Ramachandran (φ,ψ) backbone torsion angle distribution in crystal structures and low-energy Rosetta models and determined those for which the two were most in disagreement. The largest differences were found in the ψ angle distribution in β-sheets (Supporting Information Fig. S1). In crystal structures, the ψ angle distribution in β-sheets for most residues has a single peak centered around 140°; lower values of ψ are disfavored due to steric repulsion between Cβ and O (Fig.3). However, for polar and charged residues with short sidechains, Asp, Asn, Thr, and Cys, there are two distinguishable peaks in the ψ distribution in β-sheets, one around 120° and the other around 140°∼ 150°. The peak around 120° arises because the steric repulsion between Cβ and O is compensated by a hydrogen bond formed between the sidechain and the backbone amide of residue *i* + 2, which constrains the backbone ψ angle to be lower than 120° [Fig. 3(B)]. The correlation between this hydrogen bond and ψ is evident in crystal structures, especially in the loop region as shown in Figure 4. A peak in distribution arises at ψ around 120° and distance below 3 Å between Asn Oδ_1_ and N of residue *i* + 2.

Rosetta models the ψ distribution well for most residues (Supporting Information Fig. S1), reproducing the single peak around 140° and matching the intensity. However for Asn and Asp, Rosetta underestimates the population around 140°, while oversampling the region with ψ < 120°. The discrepancy between Rosetta models and crystal structures is due to energy overcounting. In the Rosetta forcefield, this conformation is favored by three energy terms, the hydrogen bond potential, the Ramachandran potential, and the backbone-dependent rotamer potential. The favorable Ramachandran and rotamer potentials both reflect the frequent occurrence of the hydrogen bond in crystal structures, and this double counting results in overstabilization of the ψ < 120° conformation. A clear artifact is that the Asn conformation is still favored even when the hydrogen bond is not formed. This leads to a correlation between the atom pair distance between Asn Oδ1 and N of residue *i* + 2 at over 4 Å and the ψ angle < 120° in β-sheets [Fig.4(D)]. That the origin of this artifact is the overcounting is confirmed by the observation that when Ramachandran potentials of Asn and Asp are substituted with those of Ala, the peak of ψ below 120° disappears.

We corrected for this double counting by iteratively correcting both the Ramachandran potential and the rotamer potential as described in the Methods. As two terms are being modified, there could be multiple independent ways to achieve the same net correction—to resolve this degeneracy, we aimed for corrections which made both the backbone torsion potential and the rotamer potential more similar to those of other residues. With the corrections, the backbone torsion potential for Asn becomes more similar to those of nonpolar residues, with the energy in the ψ < 120° region less favorable [Fig.3(E)]. The rotamer potential also no longer favors the χ_2_ ∼ 15° region when ψ is less than 120°, becoming less backbone dependent. With the optimized potential, the sidechain-backbone hydrogen bonds in the ψ < 120° still form, but they are only favored by the hydrogen bonding potential. The backbone torsion angle distributions in the modeled structures using the optimized potentials are more similar to the crystal structure distributions (Figs. 3 and 4) than those using the original potentials.

### Coupling between Lennard-Jones interactions and sidechain torsion potential

**Figure 5:**
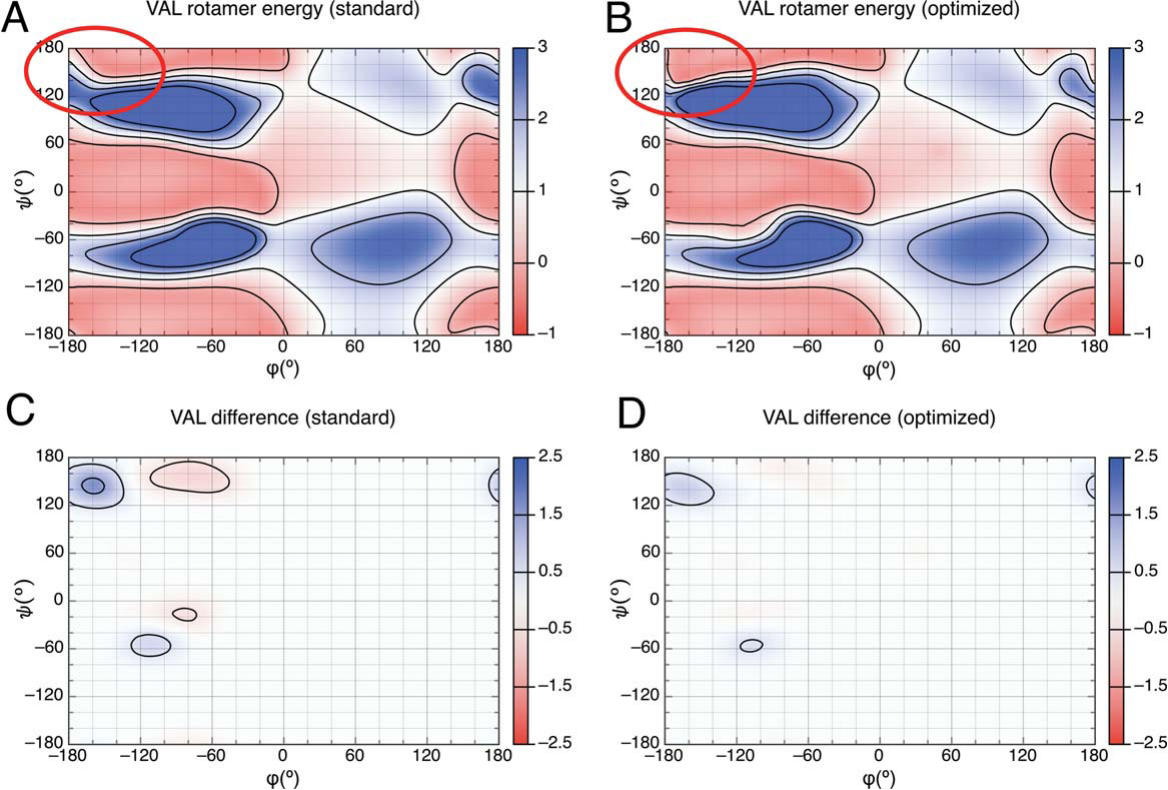
Comparison of the φ/ψ dependent sidechain torsion angle potential of Val at χ_1_ ∼ −60° (**A**) calculated from the standard library^24^ and (**B**) after the optimization. The regions showing significant difference between Rosetta models and crystal structures are highlighted with red circles. The differences in the φ/ψ-dependent sidechain torsion angle distribution between crystal structures and low-energy models are shown using (**C**) the standard Rosetta forcefield and (**D**) optimized forcefield. The color scales show the rotamer potentials (A,B) and the scaled differences in rotamer distributions (C,D).

Comparison of sidechain rotamer distributions between low-energy Rosetta models and crystal structures shows that the distributions of rotamers at χ_1_ ∼ −60° are underpopulated in Rosetta models for φ < −120° (Fig.5). Examples of this discrepancy are shown in Figure 5 for Val (χ_1_ ∼ −60°). Structural analysis shows that as φ shifts below −140°, the backbone carbonyl group of residue (*i* − 1) moves toward the sidechain, leading to steric repulsion. This clash is small, so the repulsion does not completely disfavor rotamers with χ_1_ ∼ −60°, as hey are still observed in crystal structures, but this sidechain rotamer is significantly more depleted in low-energy Rosetta models (the rotamer is observed in 20∼30% of crystal structure positions with a φ < −140°, whereas in low-energy Rosetta structures, the frequency is <5%).

Double counting energy contributions are again likely to be the origin of the discrepancy: both the rotamer potential and the Lennard-Jones potential disfavor rotamers with χ_1_ ∼ −60°, when φ is less than −140°. To remove the double counting, the rotamer potential was optimized using the iterative protocol described in the Methods section to effectively subtract the Lennard-Jones contribution from the rotamer potential. As shown in Figure5, the energy at φ < −140° is unfavorable in the original rotamer potential. After optimization, for the same ψ, the potential at φ < −140° and φ > −140° is now similar for the χ_1_ ∼ −60° rotamer [Fig. 5(B)]. With the corrected rotamer potential, the χ_1_ ∼ −60° rotamers of Val are now properly populated; the distribution in low-energy Rosetta models is now much closer to the crystal structure distribution (Fig. 5).

Additional errors were found in Tyr and Phe for which χ_2_ near 0 is overpopulated in the low-energy Rosetta models. For example, for buried positions, the Phe rotamer at χ_1_ = –70° and χ_2_ = –14° has an occupancy of 8% in crystal structures but over 20% in Rosetta models. The origin of the discrepancy here is more difficult to identify because the rotamer distribution varies little with all Rosetta energy terms up- or down-weighted individually. However, for exposed Phe with more than 20% solvent exposure, this rotamer is 9% occupied in Rosetta models, matching the rotamer library well. This suggests that packing of the rest of the protein suppresses rotamers with χ_2_ near 90° and enhances the probability of rotamers with χ_2_ near 0°. After optimizing the rotamer potential using the iterative protocol, the energy of the χ_2_ near 0 rotamers increases significantly so that the correct rotamer distribution is now recovered in Rosetta models.

### Impact of potential function corrections on the overall energy landscape

**Figure 6:**
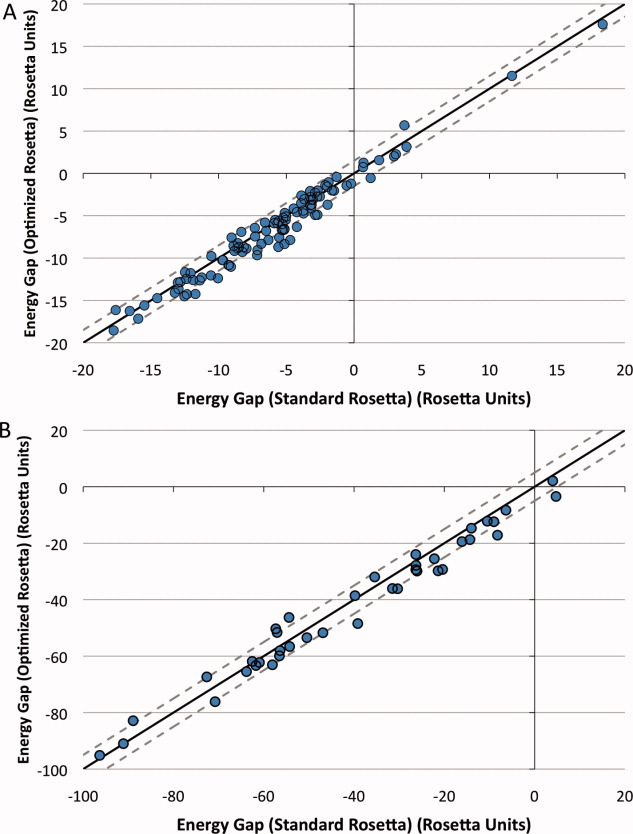
Comparison of energy gap between the native and non-native structures with optimized forcefield versus standard Rosetta forcefield (1 Rosetta energy unit ≍ 0.5 Kcal/mol). A more negative energy gap corresponds to a better discrimation between the native and decoy structures. (**A**) 110 proteins from the dataset that the forcefiled optimization is carried out on and (**B**) 55 proteins in the CASP8 benchmark set. For each protein, 10,000 full atom structures were generated with Rosetta refinement protocol using the standard or optimized Rosetta forcefield, and energy gaps between the lowest native and non-native conformations were compared as described in the Methods section. For each protein in the sets, the energy gap with the optimized forcefield is plotted against the energy gap with the standard forcefield. The dashd lines indicate that energy gap change by 1.5 energy units (A) or 5 energy units (B). Although the energy gaps are quite similar, for most difficult discrimination problems (energy gaps close to 0), there is small but consistent improvent with the optimized forcefield. [Color figure can be viewed in the online issue, which is available at wileyonlinelibrary.com.]

One of the key challenges in forcefield optimization is to prevent over-optimizing toward one set of measurements, while worsening those on others. We tested the combined changes described above by generating with the new forcefield extensive energy landscapes for each of the 110 proteins in our test set. The energy gap between near-native structures and low-energy computed structures was calculated as described in the Methods section. For each protein, the energy gap with the optimized forcefield is compared to that with the standard forcefield in Figure6. The overall impact on the energy landscape is small, on average the energy gap is shifted by 0.5Rosetta energy unit (1 Rosetta energy unit ∼ 0.5 Kcal/mol) in favor of native structures. Of 110 proteins tested here, in 19 cases, the changes in the energy gaps now favor the native structure more by 1.5 Rosetta units. No protein has an energy gap changed by more than 3 energy units. Thus, the optimization of the forcefield does not significantly alter the energy difference between native and non-native structures, while improving the geometry.

### Independent benchmark

The optimized Rosetta forcefield was applied to an additional independent benchmark, using 55 protein structures from the CASP8 experiment^25^ as described in the Methods section [Fig.6(B)]. The optimized forcefield has a small impact on the energy gaps of these proteins overall. On average the native structures are more favored by 1.4 Rosetta energy units. There are five proteins that have energy gaps > −10 Rosetta units, where the discrimination between native and decoy structure is poor. All of these proteins show improvements over 2 Rosetta units favoring the native structure.

## DISCUSSION

We show how comparison between distributions of structural features in crystal structures and low-energy computed structures can be used to guide forcefield optimization. Errors in the forcefield are detected in regions where the distributions in low-energy models differ from those in crystal structures. We apply the method to resolving the interdependencies between sidechain and backbone torsion potentials and backbone and sidechain hydrogen bonding interactions, and the approach also motivates explicit treatment of Cα hydrogen bonds. The new forcefield yields minima with improved geometry without significantly changing the position or relative depth of minima on the overall energy landscape. The approach is physically based and structure guided and can be applied more generally to improve forcefields using information from macromolecular structures.

It is instructive to compare the approach to correcting torsion potentials described here to the derivation of torsion potentials from quantum chemistry energy calculations on small peptides in MM forcefields. In both Rosetta and MM forcefields, the torsion potential is something of a catch-all for energy contributions not contained within the other more easily parameterizable terms in the potential. MM methods parameterize the torsion potential by computing the total energy of small peptides for varying values of the torsion angles and then subtract the contribution of all other terms—the remainder is the torsion potential. Likewise, we optimize the torsion potential to reproduce the correct distribution keeping other terms fixed. Both approaches have strengths and weaknesses; the QM approach has the advantage of parameterizing on energies rather than frequencies, but the disadvantage that the energy calculations can only be done on very short peptides and not in explicit solvent, while the distributions are collected from proteins of all sizes in real water. A further parallel is the adjustment of the backbone torsion potential in going from AMBER94 to AMBER99SB to favor β-strand forming peptides^13^ and sidechain torsion potential improvement in AMBER99SB-ILDN 37; our approach has the advantage of using samples spread throughout the energy landscapes of large numbers of proteins.
