# Supplementary material for: Structure-guided forcefield optimization
Source: Proteins. 2011 Feb 15;79(6):1898–909. doi: 10.1002/prot.23013 (PMC3457920; doi:10.1002/prot.23013)
Supplement: Supplementary file 1 [file prot0079-1898-SD1.pdf]

Supplementary Material

Structure guided forcefield optimization

Yifan Song[1], Michael Tyka[1], Andrew Leaver-Fay[1,†], James Thompson[1], and David Baker[1,2,]*

1. Department of Biochemistry, University of Washington, Seattle, WA 98195, USA

2. Howard Hughes Medical Institute, University of Washington, Box 357370, Seattle, Washington 98195, USA

† Present address: Department of Biochemistry, University of North Carolina, CB 7260, Chapel Hill, North Carolina 27599, USA

*To whom correspondence should be addressed. E-mail: dabaker@u.washington.edu

Supplementary Material

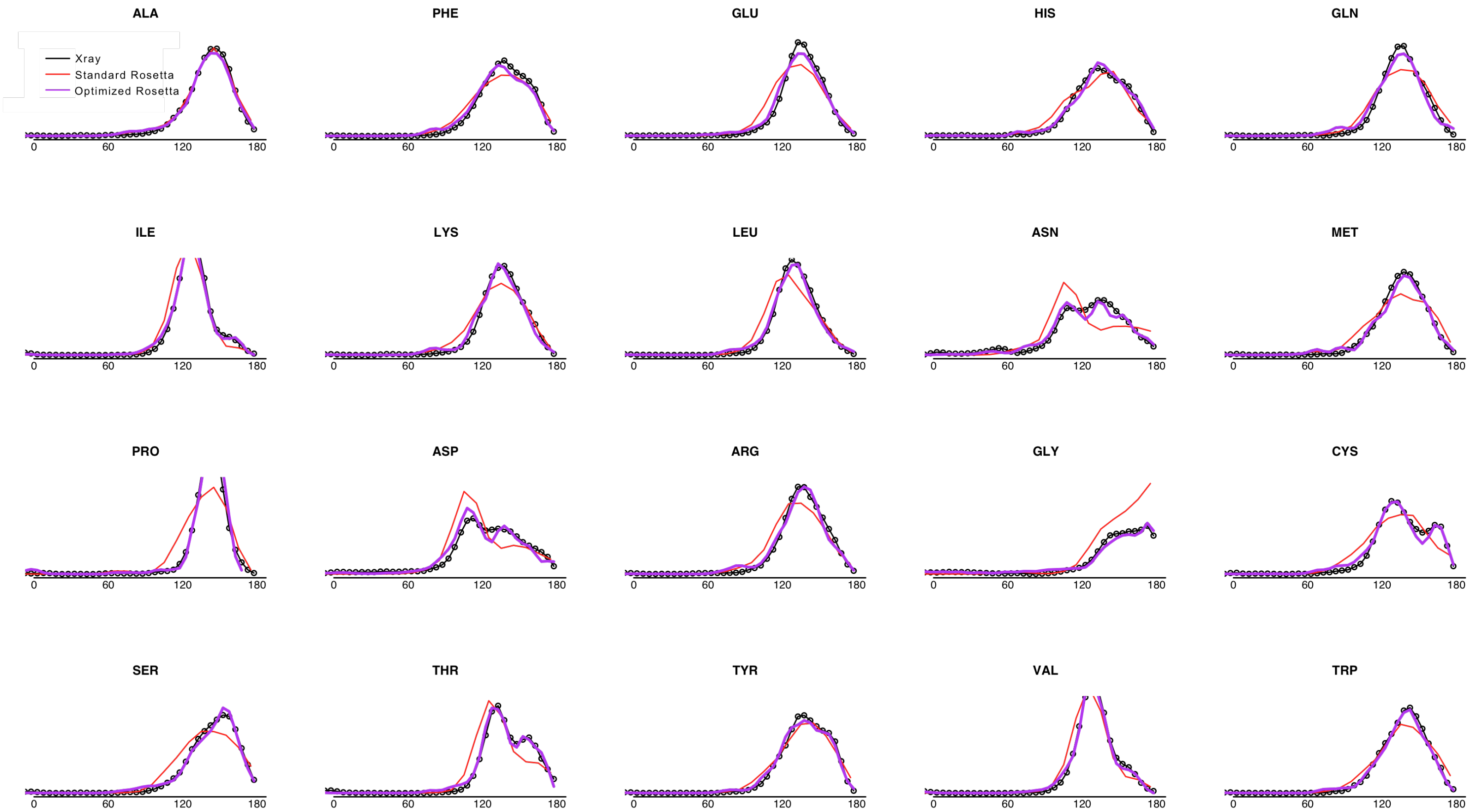

Supplementary Figure S1. The backbone ψ distribution of residues in β sheets. Black line, from xray structures; red, from standard Rosetta models; magenta, using optimized forcefield.
